# Supplementary material for: How investment in children shape fertility choices of families: Evidence from Pakistan
Source: PLOS Glob Public Health. 2024 Dec 31;4(12):e0003957. doi: 10.1371/journal.pgph.0003957 (PMC11687898; doi:10.1371/journal.pgph.0003957)
Supplement: Attachment — Submitted filename: Response to Reviewers.docx [file pgph.0003957.s001.docx]

We thank the reviewers for thorough assessment of the manuscript and insightful comments. In some cases, we made few errors and accept the changes that were given by the reviewers. We hope and have addressed the comments in best of the our abilities in the table below.

| Reviewer | Response |
| --- | --- |
| **Reviewer 1 comments** | |
| 1. I would urge the authors to conduct a broader literature review to discuss current research and how theirs contribute to the overall topic. | We appreciate your input and have considered your advice. We have conducted a thorough literature review and integrated additional references to support our arguments and contribute to the broader discussion on the topic in text lines 92, 92-95, 113, and 118. |
| 2. I am concerned about the authors use of two separate surveys. There is no mention in the method if these two surveys can be combined (or previously have been). They state that they have been conducted on the same household. Does that mean that they both have the exact same sample size and participants? More information is needed as there are concerns about the validity. | We have used a single survey i.e. Pakistan Social and Living Standards Measurement (PSLM) Survey/ Household Integrated Economic Survey (HIES) 2018-19 which offers disaggregated data to provide insights into the socio-economic conditions at the provincial level across Pakistan as mentioned in line 147 of the text.  It's noteworthy that the HIES has been incorporated into the PSLM survey since 1999. The core components and questions related to the economic aspects of the HIES were preserved within the PSLM framework mentioned in the text lines 141-143. |
| 3. In the objective, the authors state that they are looking at the education of older children. However, &ldquo;older children&rdquo; is never defined. I would urge the authors to define this population. Moreover, I would also recommend the authors to consider their study population as girls as 15 could be considered &ldquo;older children&rdquo;. Moreover, can a girl age 15 have an older child? | The idea was to highlight children who are of school age. We understand stating old age is confusing, so we have corrected it in the draft that children of school going age. This is mentioned on the draft on the following lines: 68, 128, 169, 208, 227, 262-263.  This is an interesting proposition that could be considered for future studies, especially given the prevalence of early-age marriages in Pakistan. However, our current scope is focused on assessing the impact of spending on children within a family context. While considering the wife or mother as a child was not within the scope of our study, it could potentially be explored as a separate measure in future research endeavors. |
| 4. The authors need to more clearly define their participants. For example, were women who did not have children excluded from the sample? What about women who did not answer the outcome question? I would urge the authors to a flow diagram of how they stratified the sample. | The study includes all married women within the reproductive age group, regardless of whether they have children. Women without children were not excluded from the study sample. However, the education expenditure was estimated only for women whose children are currently enrolled in school. |
| 5. Please define &ldquo;current contraception use&rdquo;. What types of contraception does it include? | Current contraception use typically refers to the practice of actively using a contraceptive method or device at the time of data collection. The common types of contraception in the PSLM survey include   i. Modern methods i.e. IUD, Injections, implants, female sterilization, male sterilization, Pills and Condoms ii. Traditional methods i.e. Withdrawal, rhythm and any other.   Included in the manuscript text lines 159-164. |
| 6. There should only be one independent variable. From my understanding of the objective, it is education expenditure. This is the most concerning issue of the manuscript. | Our primary focus has been on education expenditure. However, to conduct a more comprehensive analysis and gain insights into the associations with demographic factors, we also incorporated assessments of the wealth index and number of children.  Additionally, other variables were included in the analysis to control for potential confounding factors and ensure unbiased coefficients in the regression analysis. The inclusion of all relevant variables helps to mitigate the influence of extraneous factors, enhances the robustness of the results, and makes coefficients unbiased. |
| 7. Please define all variables in the analysis. | Current contraceptive use is defined as the active utilization of a contraceptive method or device at the time of data collection. Please refer to comment 5 for a detailed explanation. Education expenditure per child is the average amount spent on education of all the children in a family, who are in the age bracket of 4 to 25 and enrolled in school.  Mother’s employment status indicates the current employment situation of each woman, categorized as employed, unemployed, or not in the labor force/inactive.  Mother’s education level is classified into primary (1 to 5 years of education), middle (6-8 years of education), secondary (8-10 years of education), and higher education (above 10 years of education).  The number of children refers to the total count of children born to each mother. The categories range from no children to having more than five children.  The variable province and region specify the geographic location of the household within Pakistan, including regions such as Punjab, Sindh, Khyber Pakhtunkhwa, Balochistan, etc., categorized by rural or urban areas. Also included in the footnote of the text line 164. |
| 8. Please provide some citations and evidence as to why certain variables were chosen as controls. I would urge the authors to create a DAG to demonstrate how the controls were determined. I am concerned about the validity of the regression given this lack of information. | We have included references from the literature that encompass key variables essential for measuring the impact of contraceptive use. The variables selected for inclusion in the regression analysis are widely recognized in the literature for their direct correlation with contraceptive use. By incorporating these variables, we have ensured that our analysis aligns with established research findings and theoretical frameworks. The literature references for these variables have been integrated into the manuscript from text lines 166-170. We believe that the addition of literature referencing the variables appears to be adequate. |
| 9. Please note which variables were used in the wealth index and how it was calculated | The questionnaire sections used to construct the wealth index are mentioned in the text line 176-178. The process of constructing the wealth index is elaborated upon and included in the draft, spanning from text lines 175 to 183.  The variables used (text line 178-179) following the DHS methodology, are derived from the reference. |
| 10. Please provide more information on how the regression was developed and executed. | We have used logistic regression since our dependent variable is binary. The explanatory variables were selected based on the literature which shows a significant impact on the dependent variables, mentioned in the text lines 234-239. |
| 11. It is unclear what the quality-quantity preference of contraceptive users is. I would urge the authors to simplify their analysis to only the regression. | Overall, the preference for quality over quantity is evident, particularly among the middle and upper-middle classes, where a trend towards smaller families is observed as opposed to larger ones.  Our regression analysis reveals that the likelihood of contraceptive use increases with the number of children across all three models. Additionally, we find that the number of children decreases as families transition from the poorest to the wealthiest quintile.  Education expenditure is correlated with the wealth index, indicating that wealthier households have the resources to afford their children's education expenses, despite having more children. It was crucial to assess different income classes to determine whether the impact is consistent across different socio-economic groups.  Moreover, there are notable differences in behavior between urban and rural settings. The relationship between income and family planning behavior varies among families. The inclusion of the quality-quantity aspect aimed to emphasize that the middle and upper-middle classes have a higher probability of using contraceptives, thereby reducing fertility. However, this trend does not uniformly increase across income classes in urban and rural settings. Interestingly, contraceptive use is not significantly higher among the wealthiest households in Pakistan compared to the upper-middle class. |
| 12. It is unclear why families with no children were included in this analysis. How can you have education expenditure if you do not have a child? Please provide a justification | Families without children naturally had zero expenditure per child since there were no children to allocate expenses to. We chose to include families without children in our analysis to ensure a comprehensive assessment of married women of reproductive age and to thoroughly analyze the impact on contraceptive use across the entire population. Excluding this subgroup would skew our coefficients, as we aimed to assess one group (spenders) against all other groups, ensuring accurate estimates at the national level. |
| 13. The last child sex in Table 1 is unnecessary. | The variable has been removed from Table 1 as suggested. |
| 14. Please provide an x-axis label on all of the graphs | The X-titles have been added to all the graphs (Figure 1, Figure 2, and Figure 3) |
| 15. I am very concerned that the mother’s age was not a considered variable in the regression as this would clearly impact her ability to have children. | The mother's age variable as control was included in the regression but not incorporated in Table 1. It is mentioned in the notes of the table. However, we have added the mother’s age variable in Table 1 as suggested. |
| 16. Rather than categorizing expenditure, the authors should consider a linear regression | Due to the scattered variability of the variable, characterized by a higher standard deviation, it was deemed necessary to create categories based on the varied distribution of the data. |
| 17. Please only report the outcome variable in the regression. | While we understand your preference for focusing solely on the outcome variable in the regression results, we believe that including additional variables in the text provides valuable context and insight into the factors influencing the outcome. These variables align with existing literature, demonstrating the robustness of our regression model. Their inclusion allows for a more thorough interpretation of the results and enhances the overall understanding of the relationship between the variables. |
| 18. I would urge the authors to do a broader literature review for the discussion to compare and contrast their findings. | Updated as suggested. The additional references in support and comparison of the results are added in the text lines 265, 267, 270-273, 274, 277, and 298. |
| 19. Please provide strengths and limitations | The limitation section has been included in the draft as suggested. Please refer to the text lines 308-317. |
| **Reviewer 2 comments** | |
| The writing is intelligible, but there are some instances where the language is a little ambiguous and a few instances of grammatical or English language errors. This is only a matter of copyediting however, which I would encourage the authors to review by engaging a native English speaker on their review. Some examples of this include (but are not limited to):  - line 268-269: &#39;These women tend to have more children than they would have if they been working&#39;, which needs correcting to &#39;These women tend to have more children than they would have if they had been working&#39;  - line 277: &#39; These affluent and educated Pakistani mothers may be finding that labor market options are not commiserate with their education or expectations...&#39; which needs correcting to &#39; These affluent and educated Pakistani mothers may be finding that labor market options are not commensurate with their education or expectations. | Revised as suggested. |
| The tables and figures are very useful, but would benefit from better labelling for improved interpretability. For example:  - Figure 1 needs a label on the X axis, and a reference to the unit of measurement on the Y axis (PKR-units whenever needed)  - Figure 2 needs a label on the X axis, and a reference to the unit of measurement on the Y axis (assume it is CPR, but this isn&#39;t fully clear)  - Table 3 of the regression outputs is quite hard to interpret for an everyday reader. This could be moved to an annexe.  - Figure 3 needs units on the X axis label, and clearer/more visible labels (they&#39;re a bit hard to see). | Thank you for your constructive feedback on the clarity of our tables and figures.  Figure 1: We have revised the figure and ensured the labels and units of measures are added.  Figure 2: we have added a label on the X-axis and we have used contraceptive use on the Y-axis.  Table 3: The coefficients are important to include to interpret the relationship between the dependent variable (DV) and independent variables (IVs). Given their significance, we would prefer to retain the regression outputs in the main draft.  Figure 3: We have updated the units on the X-axis label and ensured that the labels are clearer and more visible for ease of interpretation as suggested. |
| It would be helpful if the research&#39;s main hypothesis / research question were more prominent in the abstract. I would suggest adding this to the last line of the introduction in the abstract. This would really help with clarity. | Revised as suggested in the text line 45-47. |
| Similarly the title is a little ambiguous and could better reflect the hypothesis of the research. I would suggest it more specifically reflect the research question being investigated, for example &#39;Does investment in children(&#39;s education) play a role in fertility preferences in Pakistan?&#39;, or &#39;Does investment in children have a bearing on family size and contraceptive use in Pakistan?&#39; etc. Currently it&#39;s not very clear from the title what the article is about. | The title has been revised as suggested. |
| The policy implications of the findings are not well articulated, especially for the wealthiest quintiles (which diverges from the overall trend). It would be interesting to compare with other contexts how these phenomena have influenced policy and decision making elsewhere. | Our primary objective was to shed light on societal behavior changes within the context of Pakistan, particularly in relation to fertility theories. We aimed to determine whether these changes align with established fertility theories and to provide insights for future research where the education sector will become a prospect of research, making it beyond the scope of this article. |
| Additionally, the point that increased CPR correlates with the sex of the last child being male is interesting, and I think warrants being acknowledged further than one line. It would be interesting to hear the authors&#39; policy recommendations related to this. | As this variable serves as a control in our analysis, we have not delved into it extensively in this paper. However, we have conducted related work in this domain, and we provide the citation for your reference:  *Khan, A. A., Hussain, H., Amir, R., & Shujaat, K. (2021). Do boys complete the family: Sex selection by choosing when your family is complete? Literacy (years of education), 3(3), 0-002.* |
| Lastly, the research could benefit from a limitations section. This could include, for example, that the data used only surveys married women; two surveys are used, albeit of the same households, but the data from each cannot be matched to specific households; and that the data is only indicating correlation, and what this means for its interpretation (etc). | We have used a single survey i.e. Pakistan Social and Living Standards Measurement (PSLM) Survey/ Household Integrated Economic Survey (HIES) 2018-19 which offers disaggregated data to provide insights into the socio-economic conditions at the provincial level across Pakistan as mentioned in line 150 of the text. The expenditures taken are for the same households.  It's noteworthy that the HIES has been incorporated into the PSLM survey since 1999. The core components and questions related to the economic aspects of the HIES were preserved within the PSLM framework mentioned in the text line 141-143.  Our study focuses primarily on investment in children's education as a proxy for quality in family planning decisions. While education is undoubtedly an important determinant, other factors contributing to the quality of children, such as access to healthcare, nutrition, and social support, are not disaggregated for household members. Therefore, our findings may provide an incomplete picture of the quality-quantity tradeoff in fertility preferences. The limitation section is added in the manuscript from text line 308-317. |
