# Supplementary material for: How investment in children shape fertility choices of families: Evidence from Pakistan
Source: PLOS Glob Public Health. 2024 Dec 31;4(12):e0003957. doi: 10.1371/journal.pgph.0003957 (PMC11687898; doi:10.1371/journal.pgph.0003957)
Supplement: Attachment — Submitted filename: comments_sarashwati Giri.docx [file pgph.0003957.s002.docx]

Clarify Hypothesis and Objective: Explicitly state the hypothesis and main objective in the abstract to make it clear from the outset what the study aims to demonstrate.

Highlight Key Findings: In the conclusion, briefly emphasize the most significant findings and their implications for family planning policies, especially focusing on the observed exceptions in wealthier rural households.

Methodology

Clear and Detailed: The methodology is clearly described and well-structured, providing necessary details on the data sources, sampling, and analysis.

Comprehensive Analysis: Appropriate use of multinomial logistic regression and control for various factors enhances the study's reliability.

Discussion

strong Theory Integration: Effectively ties findings to Becker's theory of fertility and global trends.

Policy Relevance: Highlights key policy implications for education and employment opportunities for women.
