# Supplementary material for: How investment in children shape fertility choices of families: Evidence from Pakistan
Source: PLOS Glob Public Health. 2024 Dec 31;4(12):e0003957. doi: 10.1371/journal.pgph.0003957 (PMC11687898; doi:10.1371/journal.pgph.0003957)
Supplement: Attachment — Submitted filename: Response to Reviewers.docx [file pgph.0003957.s003.docx]

We thank the reviewers for thorough assessment of the manuscript and insightful comments. In some cases, we made few errors and accept the changes that were given by the reviewers. We hope and have addressed the comments in best of the our abilities in the table below.

| Reviewer | Response |
| --- | --- |
| **Editor’s comments** | |
| In addition, we suggest that you provide further analysis of the theory of fertility that "posits that with increasing wealth, families value children for themselves (and therefore invest in their futures) rather than as productive assets" in light of changing lifestyles and primary sources of income in Pakistan. The possibility should be considered that families are willing to pay for education to improve long-term income-earning potential of children as a long-term investment in the families' income and wealth.  In addition, as noted by Reviewer #3, income-earning potential of women in the labor market should be addressed. The discussion and conclusion section of your manuscript would be strengthened by adding this additional analysis.  In addition, before submitting your revised manuscript, we suggest that you work with a copyeditor to improve the overall readability of your manuscript. | Thank you for your valuable suggestion. We have expanded the analysis as suggested. The results have been included in the Results section (lines 247-257) and further discussed in the Discussion section (lines 296-298). These additions focus on how rising wealth influences families to prioritize children's education and healthcare as a long-term investment, aligning with the fertility theory.  We have addressed the income-earning potential of women in the labor market by conducting additional analysis to compare the income of family planning users and non-users (lines 247-257). We have discussed this in the Discussion section (lines 338-343) and expanded on it in the Conclusion (lines 367-368) to emphasize the importance of equitable labor market opportunities for women in shaping fertility preferences.  We also have improved the overall readability of the manuscript through copyediting. |
| **Reviewer 3 comments** |  |
| Clarify Hypothesis and Objective: Explicitly state the hypothesis and main objective in the abstract to make it clear from the outset what the study aims to demonstrate.  Highlight Key Findings: In the conclusion, briefly emphasize the most significant findings and their implications for family planning policies, especially focusing on the observed exceptions in wealthier rural households. | The abstract has been revised to clearly state the hypothesis and the main objective is now explicitly outlined, focusing on assessing the relationship between affluence, education spending, and family planning in Pakistan (lines 45-48).  We have revised the conclusion to highlight the key findings (lines 70-76). |
| **Reviewer 4 comments** |  |
| Abstract: Methodology: How did study authors get access to the data used for this study | Revised as suggested in lines 48-52 and 68-74. |
| Introduction: - Lines 82-85: I would recommend authors consider the influence of innovation in terms of improvement in maternal and child health and impact on child and mother mortality - Lines 91-94: I would expect that as the number of children decreases, investment of children and a direct increase on reserves (finance). The sentence at its current state isn’t clear | The introduction has been expanded to include a discussion of how innovations in maternal and child health, such as improved healthcare delivery and diagnostics, have contributed to the reduction in mortality. These improvements are linked to changes in fertility preferences (lines 93-98).  The sentence as written is intended to highlight the tradeoff between the number of children and the costs associated with raising them, as proposed by Becker. We are not analyzing the direct increase in financial reserves or savings as a result of having fewer children. Instead, the focus is on how families shift from having many children to investing more resources in fewer children, which leads to the "quantity to quality" tradeoff. Our analysis links the cost of raising children to the decision to have fewer of them, without exploring the financial savings aspect. |
| Methodology: - Setting and the Survey: How did authors access this data for analyses? - Authors should consider providing more details on how data was abstracted from the database. | The Methodology section now clearly explains that secondary data was accessed from the Pakistan Bureau of Statistics official website for analysis (lines 151-157). |
| Data analysis: How were the characteristics of study participants gotten? Was the data set weighted after extracted from the database? I have seen how this was done. | The Methodology section has been updated to explain that the participant characteristics were obtained directly from the PSLM and HIES datasets, which were already weighted to reflect the population distribution. This ensures that the analysis accurately represents the national population (lines 159-160). |
